# Neuroprotection: Rescue from Neuronal Death in the Brain

**DOI:** 10.3390/ijms22115525

**Published:** 2021-05-24

**Authors:** Bae Hwan Lee

**Affiliations:** Department of Physiology, Brain Korea 21 PLUS Project for Medical Science, Yonsei University College of Medicine, Seoul 03722, Korea; bhlee@yuhs.ac

The brain plays important roles in mental processing and in controlling other bodily organs. However, the brain tissue is vulnerable to exogenous or endogenous injury. After injury, neuronal death leads to functional deficits and neurological disorders. Therefore, protection from neuronal death is crucial for preserving brain functions.

This Special Issue aimed to understand the mechanisms underlying neuroprotection and to explore potential therapeutic strategies for recovering brain functions from neurological disorders. In this Special Issue, nineteen papers were published, including thirteen original articles and six review papers dealing with interesting subjects which are related to different brain functions and disorders.

Stroke including ischemia is one of the major villains responsible for neuronal death and mortality. A review by Dong et al. [1] summarized the current understanding of the mechanisms of pathogenesis in ischemic stroke and the updated developments of nanomedical therapeutics such as nanoparticles, liposomes, and cell-based nanovesicles. It has been shown that cation-chloride cotransporters (CCCs) are expressed in neurons and contribute to numerous physiological functions. In a review paper, Josiah et al. [2] introduced the recent advances in the functional regulations of the CCCs and their signaling pathways in stroke. Using a rodent model of global cerebral ischemia, Kang et al. [3] observed that amiloride, an inhibitor of the sodium–hydrogen exchanger-1, attenuates hippocampal injury by reducing zinc accumulation in mice, and Hong et al. [4] reported that administration of N-acetyl-L-cysteine, an antioxidant, reduces neuronal death induced by cerebral ischemia and over-activation of transient receptor potential melastatin 2 (TRPM2) channels in rats. Jung et al. [5] described that pyridoxine (a form of vitamin B6) deficiency increases neuronal death after ischemia by enhancing serum homocysteine levels and lipid peroxidation, and by reducing nuclear factor erythroid-2-related factor 2 (Nrf2) levels. Pyridoxine also decreases regenerative potentials by reducing brain-derived neurotrophic factor (BDNF) levels in the hippocampus of gerbils. Markosyan et al. [6] reported that preventive triple gene therapy by adenoviral vectors carrying genes encoding vascular endothelial growth factor (VEGF), glial cell-derived neurotrophic factor (GDNF), and neural cell adhesion molecule (NCAM), or by gene-engineered umbilical cord blood mononuclear cells over-expressing recombinant VEGF, GDNF, and NCAM, has beneficial effects on the recovery from stroke in the brain of rats. Tanioka et al. [7] observed that intra-arterial injection of lipid emulsion during reperfusion shows neuroprotective effects in an in vivo rat model of ischemic reperfusion injury. Using primary astrocyte cultures, Mitroshina et al. [8] demonstrated that ischemic-like conditions decrease network connectivity and the stimulation with ATP under normal conditions increases the number of connections, implying that astrocytes can constitute a functional network in response to a stimulation. Using organotypic hippocampal slices of mice pups, Ort et al. [9] found that neither nimodipine (an L-type voltage-gated calcium channel blocker) alone nor in combination with amiloride (an acid-sensing ion channel inhibitor) showed any improvement in an in vitro ischemia model. The combination of both components dissolved in dimethyl-sulfoxide (DMSO) even increased cell death, although this effect was not observed in the condition treated with amiloride alone. In this regard, they suggested that DMSO should be used with caution in neuroprotective experiments. 

In relation to Alzheimer’s disease (AD) and memory functions of the brain, a review paper by Kim et al. [10] focused on the recent progression in the beneficial effects of adiponectin on AD and its potential mechanisms related to the diverse medications of adiponectin in AD treatment. Using a mouse model of AD, Gonzalo-Gobernado et al. [11] observed that intraperitoneal administration of liver growth factor (LGF) restored cognitive deficits and reduced amyloid-β (Aβ) content in the APPswe mice which over-express the Swedish APP mutation, with Aβ plaque deposits in the hippocampus and cerebral cortex. Ye et al. [12] reported that soybean-derived phosphatidylserine, a membrane phospholipid, enhances memory function in memory deficit rats by a trimethyltin, a neurotoxin affecting the brain. In a human study by Piotrowicz et al. [13], BDNF increased after exercise in normoxia and hypoxia athletes but cognitive performance was not improved by acute elevation of BDNF. Indeed, BDNF plays an important role in the growth of neurons and plasticity of neuronal synapses. The roles of BDNF in neuronal plasticity and neuroprotection were highlighted in a review by Colucci-D’Amato et al. [14]. 

Seizure deteriorates the function of the brain. Huang et al. [15] demonstrated that peroxisome proliferator-activated receptor γ coactivator 1-α (PGC-1α) activated by status epilepticus regulates the VEGF/VEGFR2 (VEGF receptor 2) pathway through mitogen-activated protein kinase kinase (MEK)/extracellular signal-regulated kinase (ERK) signaling and exhibits neuroprotective effects against neuronal death in the hippocampus of rats. In this regard, VEGF plays a crucial role as a neuroprotective factor in the nervous tissues. A review by Silva-Hucha et al. [16] summarized the contribution of VEGF to differences between the oculomotor system and other motor neurons in relation to vulnerability to degeneration.

In the brain, reactive oxygen species (ROS) increase susceptibility of the brain to neuronal damage and functional deficits. Abnormal levels of ROS are regulated by cellular defense mechanisms of antioxidants in the brain. A review paper by Lee et al. [17] presented the roles of various antioxidants in the brain and suggested the potential of antioxidants for the protection of the brain from oxidative insults. Using organic hippocampal slice cultures (OHSCs), Lee et al. [18] found that ascorbic acid (an antioxidant) treatment was effective for survival of neurons in OHSCs after kainic acid (KA) insult, but that its protective effect was different depending on aging of the OHSCs. In Parkinson’s disease (PD), oxidative stress induced by ROS is a major cause of the dopaminergic cell death. Mimicking PD-like syndrome to induce cell death through mitochondrial oxidative stress, Haque et al. [19] used neurotoxins including 1-methyl-4-phenylpyridinium ion (MPP^+^) and hydrogen peroxide (H_2_O_2_) in human dopaminergic neuroblastoma cells and found that the pharmacological inhibition by a selective antagonist of G protein-coupled receptor 4 (GPR4) or genetic deletion of GPR4 decreases apoptotic cell death through the modulation of phosphatidylinositol biphosphate (PIP_2_)-mediated calcium signaling.

As reported in many previous studies and in this Special Issue, mechanisms of neuroprotection are complex. Understanding detailed mechanisms underlying neuroprotection may lead to the development of effective therapeutic strategies for the prevention of neuronal death and for improvement of functional recovery from neural injury or neurodegeneration.

## Data Availability

Not applicable.
